# Pup Vibrissae Stable Isotopes Reveal Geographic Differences in Adult Female Southern Sea Lion Habitat Use during Gestation

**DOI:** 10.1371/journal.pone.0157394

**Published:** 2016-06-15

**Authors:** Alastair M. M. Baylis, Gabriele J. Kowalski, Christian C. Voigt, Rachael A. Orben, Fritz Trillmich, Iain J. Staniland, Joseph I. Hoffman

**Affiliations:** 1 South Atlantic Environmental Research Institute, Stanley, FIQQ1ZZ, Falkland Islands; 2 Falklands Conservation, Stanley, FIQQ1ZZ, Falkland Islands; 3 Department of Biological Sciences, Macquarie University, Sydney, NSW, 2109, Australia; 4 Department of Animal Behaviour, Bielefeld University, Bielefeld, Germany; 5 Department of Animal Ecology, University of Potsdam, Potsdam, Germany; 6 Department of Evolutionary Ecology, Leibniz Institute for Zoo and Wildlife Research, Alfred-Kowalke-Str. 17, 10315, Berlin, Germany; 7 Department of Fisheries and Wildlife, Oregon State University, Hatfield Marine Science Center, Newport, Oregon, 97365, United States of America; 8 British Antarctic Survey NERC, High Cross, Madingley Road, Cambridge, CB3 0ET, United Kingdom; Phillip Island Nature Parks, AUSTRALIA

## Abstract

Individuals within populations often differ substantially in habitat use, the ecological consequences of which can be far reaching. Stable isotope analysis provides a convenient and often cost effective means of indirectly assessing the habitat use of individuals that can yield valuable insights into the spatiotemporal distribution of foraging specialisations within a population. Here we use the stable isotope ratios of southern sea lion (*Otaria flavescens*) pup vibrissae at the Falkland Islands, in the South Atlantic, as a proxy for adult female habitat use during gestation. A previous study found that adult females from one breeding colony (Big Shag Island) foraged in two discrete habitats, inshore (coastal) or offshore (outer Patagonian Shelf). However, as this species breeds at over 70 sites around the Falkland Islands, it is unclear if this pattern is representative of the Falkland Islands as a whole. In order to characterize habitat use, we therefore assayed carbon (δ^13^C) and nitrogen (δ^15^N) ratios from 65 southern sea lion pup vibrissae, sampled across 19 breeding colonies at the Falkland Islands. Model-based clustering of pup isotope ratios identified three distinct clusters, representing adult females that foraged inshore, offshore, and a cluster best described as intermediate. A significant difference was found in the use of inshore and offshore habitats between West and East Falkland and between the two colonies with the largest sample sizes, both of which are located in East Falkland. However, habitat use was unrelated to the proximity of breeding colonies to the Patagonian Shelf, a region associated with enhanced biological productivity. Our study thus points towards other factors, such as local oceanography and its influence on resource distribution, playing a prominent role in inshore and offshore habitat use.

## Introduction

Elucidating marine predator habitat use is an important challenge in behavioural ecology because it underpins species management and conservation, and has potentially important theoretical and far reaching ecological implications [1,2]. However, knowledge of habitat use remains rudimentary for many marine predators. Although biologging tags enable marine predator habitat use to be directly assessed, financial and logistical constraints and ethical considerations associated with capture and tagging [3] often limit the number of individuals that can be tracked, which may not be representative. This is particularly problematic for central place foraging marine predators, as these often exhibit individual foraging specialization, foraging site fidelity and colony partitioning of foraging areas [4–8]. Under such circumstances, it would be desirable to study as many individuals from as many breeding colonies as possible in order to obtain an accurate representation of habitat use for a given species. Fortunately, biogeochemical markers provide powerful tools to study marine predator habitat use, in part because sample sizes large enough to assess population-level variation can be efficiently and economically gathered either remotely (for example through biopsy sampling) or from nutritionally dependant offspring that are a proxy for adults, but often easier to sample [9,10].

One of the most common biogeochemical approaches to indirectly track animals is the use of naturally occurring stable isotope ratios [11]. Stable isotope ratios of metabolically inert tissues such as vibrissae, feathers, and teeth, remain unchanged once grown. Accordingly, these tissues assimilate food-web conditions at the time of growth and can be used to indirectly track individuals over time [12]. For example, in otariids the isotope values of vibrissae represent an individual’s trophic and spatial history, with carbon stable isotope ratios (δ^13^C values) providing a proxy of foraging habitat (offshore/pelagic animals are more depleted in δ^13^C than inshore/benthic animals) and nitrogen stable isotope ratios (δ^15^N values) providing a proxy of trophic level [12–14].

Here we use vibrissae stable isotopes to elucidate the habitat use of southern sea lions (SSL) (*Otaria flavescens*). SSL experienced a precipitous decline at the Falkland Islands in the South Atlantic, with annual pup production falling from over 80,000 in the 1930s to less than 6,000 in the 1960s [15]. The population continued to decline into the mid 1990s and, although now increasing, it remains at less than 6% of the 1930s estimate [15]. Despite currently breeding at over 70 sites around the Falkland Islands (a similar breeding distribution as in the 1930s), current knowledge of SSL foraging ecology is principally derived from a single breeding colony, Big Shag Island (East Falkland). Data from biologging tags and stable isotopes showed that adult female SSL breeding at Big Shag Island forage in two discrete habitats, corresponding to inshore (coastal) and offshore waters (outer Patagonian Shelf) [16]. Adult female SSL that forage offshore travel further, dive deeper and have characteristically lower δ^13^C and δ^15^N values compared to adult female SSL that forage inshore [16].

An unresolved question is whether the inshore versus offshore pattern in habitat use described at Big Shag Island is representative of adult female SSL habitat use at other breeding colonies around the Falkland Islands. However, the majority of SSL breeding colonies are logistically difficult to access and the deployment of biologging tags is impractical because a single capture would necessitate disturbing an entire breeding colony (SSL flee when approached). Consequently, we used stable isotope analysis of pup vibrissae to elucidate the broader geographic pattern of adult female SSL habitat use across the Falkland Islands. Given that otariid pups are nutritionally dependant prior to weaning and vibrissae development occurs *in utero*, the isotope values of pup vibrissae are positively and linearly correlated with their mothers (although some fractionation exists) [9,17,18]. Hence, pup vibrissae provide a proxy for adult female SSL diet and habitat use, with differences among pups reflecting individual differences in the diet and habitat use of their mothers [9,17,18]. Given the precipitous decline of SSL at the Falkland Islands and their failure to recover, elucidating broad patterns in habitat use could be an important step toward understanding the factors that impede population recovery.

## Materials and Methods

### Ethics statement

Research was approved by the Falkland Islands Government Environmental Planning Department and conducted under permits R14/2013 and R14/2014 issued by the Falkland Islands Government.

### Sample collection

**Fig 1:**
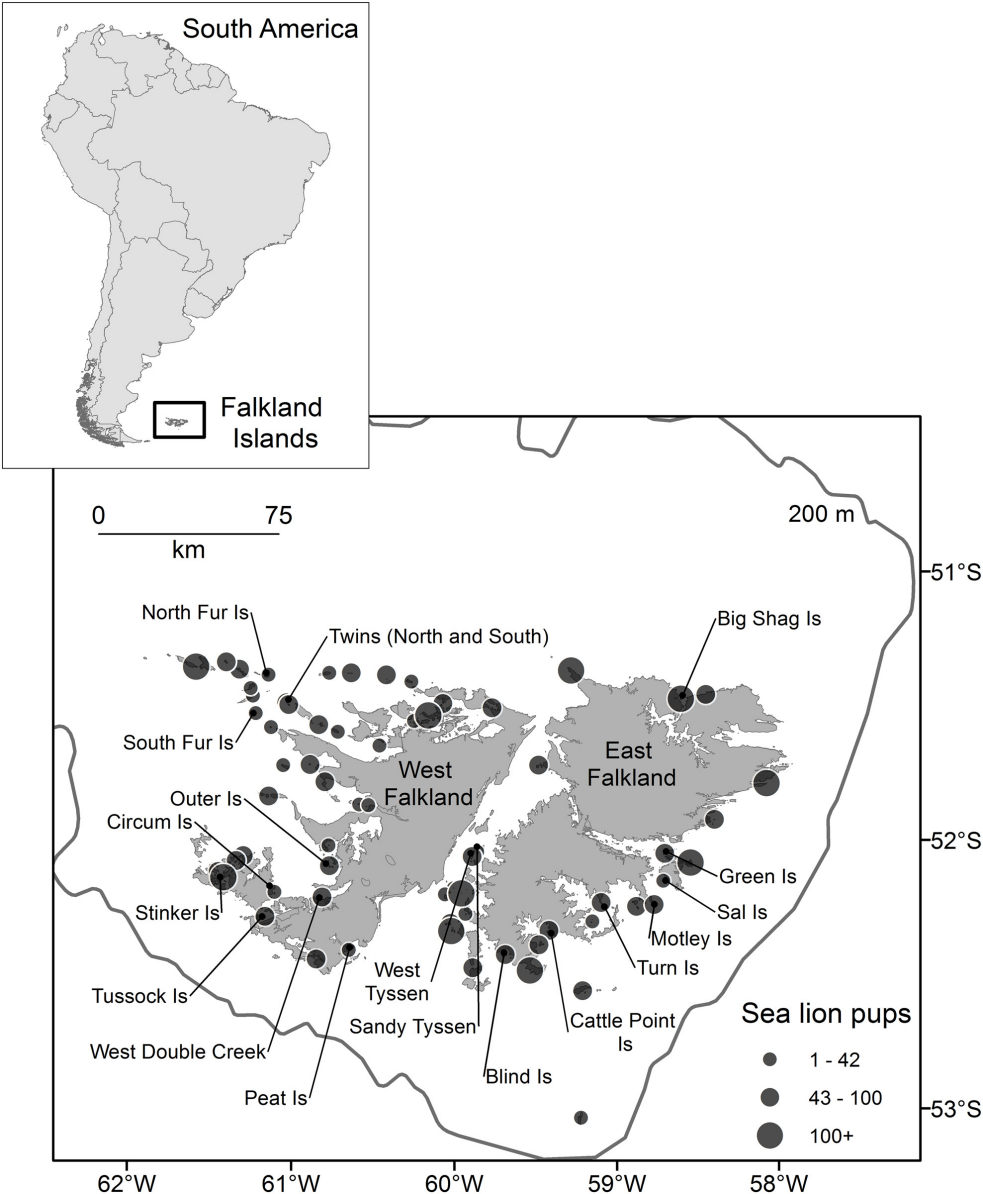
We collected southern sea lion pup vibrissae from 19 different breeding colonies across the Falkland Islands. Samples from Big Shag Island and Turn Island were collected in 2013, while the remainder of breeding colonies were sampled in 2014. Circle sizes are proportional to annual pup production, determined from direct counts in 2014 (see also Table 1) (source of 2014 Falkland Islands census data [15]). The 200 m contour depicts the edge of the Patagonian Shelf.

We initially sampled pup vibrissae in February 2013 from two breeding colonies, Big Shag Island and Turn Island (Fig 1), which were visited over an extended period (≥ 1 week). For the remaining 17 breeding colonies (Fig 1), pup vibrissae were sampled during a single visit lasting 1–3 hours between 16 January and 6 February 2014, as part of an archipelago wide census [15]. Pups were captured by hand and manually restrained. Vibrissae (*n* = 65) were sampled by cutting the largest one as close to the skin as possible with a pair of cutting pliers. The ages of the pups sampled were unknown. However, our analyses focussed on the isotopic signature of the final 1 cm of vibrissae (*i*.*e*., the distal end), the oldest vibrissae section. Although vibrissae growth rates for SSL are unknown, foetal vibrissae growth rates for Steller sea lions (*Eumetopias jubatus*) average 0.73 ± 0.05 cm/mo [19]. This suggests the final 1 cm of vibrissae integrates roughly 40 days of dietary information and is a proxy for adult female SSL foraging habitat while the pup was *in utero* [9]. We did not correct stable isotope values for mother to pup enrichment because our analysis does not attempt to reconstruct diet or directly compare values from adults to pups.

### Analysis

Stable isotope analyses were undertaken at the Leibniz Institute for Zoo and Wildlife Research, Berlin, Germany. Vibrissae were cleaned using a Chloroform-Methanol (2:1) mixture, rinsed with distilled water and then dried in a drying oven at 50°C. To produce a meaningful isotopic measurement, our target mass for each vibrissae segment was 0.5 mg. Thus, we took a sub-sample from each 1 cm section. Samples were packed in tin capsules, and carbon and nitrogen stable isotope ratios were measured using Flash elemental analyser (ThermoFisher Scientific, Bremen, Germany) interfaced in continuous mode via a Conflo to a stable isotope ratio mass spectrometer (Delta V Advantage, ThermoFisher Scientific, Bremen, Germany). Precision and accuracy of measurement was confirmed using laboratory standards. Stable isotope ratios were measured in parts per mille ‰ deviation from international standards (V-PDB for carbon and AIR N2 for nitrogen), according to the following equation δ X  =  [(R_sample_/R_standard_) –1] ×1000 where X is ^15^N or ^13^C and R is the corresponding ratio of (^15^N/^14^N) or (^13^C/^12^C). Stable isotope ratios are reported as δ^13^C values for carbon and δ^15^N values for nitrogen. Precision of measurements equalled ± 1.1 ‰ (one standard deviation) for carbon and ± 0.06 ‰ for nitrogen stable isotope ratios.

To analyse variation among pup vibrissae isotope values, we used a model-based clustering approach (based on δ^13^C and δ^15^N values) implemented within the R package ‘Mclust’ [20,21]. The clustering process estimates a model for the data that allows for overlapping clusters with varying geometric properties and that quantifies the uncertainty of observations belonging to clusters [20]. One advantage of a model-based clustering approach is that the optimal number of clusters can be selected using Bayesian Information Criterion. The ellipses produced by the ‘Mclust’ package, are the multivariate analogs of standard deviations [20]. Differences in δ^13^C and δ^15^N isotope values between clusters were tested using Welch’s ANOVA and a Games-Howell post-hoc test (to account for unequal variances), while χ^2^ tests were used to compare differences between breeding colonies in the number of pups assigned to inshore and offshore clusters.

Finally, adult female SSL breeding at Big Shag Island preferentially forage offshore, in proximity to the Patagonian Shelf slope [16]. To determine whether the proximity of breeding colonies to the shelf slope influenced inshore/offshore habitat use, we also calculated the distance of each colony to the Patagonian Shelf slope (defined here as the distance to the 200 m bathymetry contour), using ArcMAP (ArcGIS, Redlands, CA, USA). To test whether the proportion of inshore to offshore foragers differs significantly with distance to the Patagonian Shelf, we then constructed a generalised linear model with a binomial error structure in R. Statistical significance was determined using a χ^2^ test. This analysis was based on 15 colonies for which at least one individual was assigned to either the inshore or offshore cluster.

## Results

**Fig 2:**
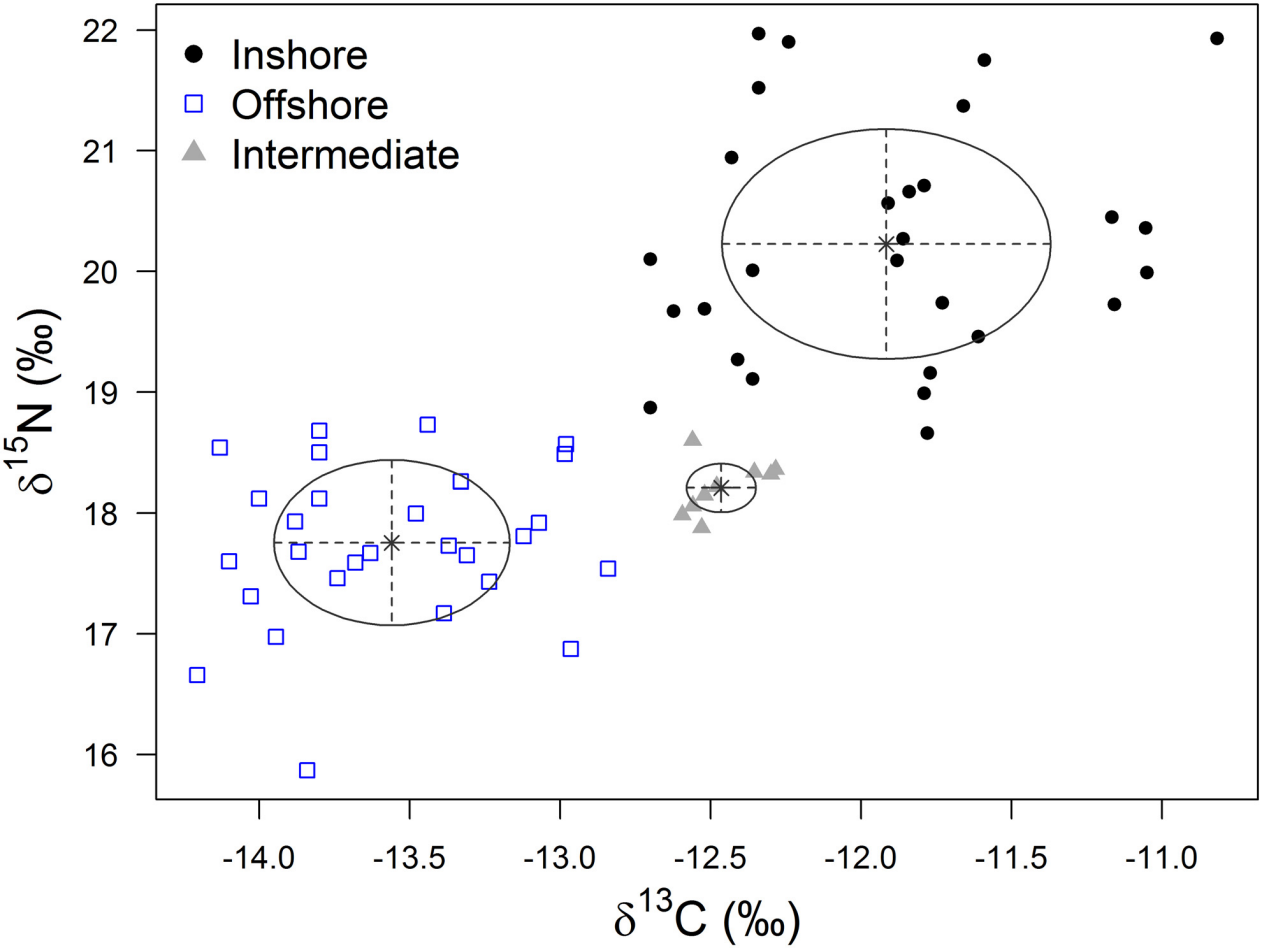
We used the vibrissae stable isotope values of 65 southern sea lion pups as proxies for adult female habitat use. Model-based clustering identified three distinct clusters (represented by ellipses, the multivariate analogs of standard deviations). These represent adult females that foraged in inshore habitats (black dots), offshore habitats (blue squares) or had δ^13^C and δ^15^N values that were intermediate to inshore/offshore values (grey triangles).

A total of 65 vibrissae samples were analysed from 19 breeding colonies around the Falkland Islands (Fig 1, S1 Table). The number of samples obtained from each breeding colony varied between one and 16, while distance to the Patagonian Shelf slope ranged from 40–110 km (Table 1). Model-based clustering revealed that individual pup vibrissae could be separated into three clusters represented by ellipses that encompassed distinct isotopic niche areas (Fig 2). The majority of samples (n = 48 or 74%) were within the quantile of lowest uncertainty (<0.75), and only four samples fell within the quantile of highest uncertainty (>0.95), lending confidence to the accuracy of the groupings (S1 Fig). Overall, δ^13^C and δ^15^N values differed significantly among the three clusters (δ^13^C: *F*_*2*,*38*.*7*_ *= * 105.9, *P* < 0.001, δ^15^N: *F*_*2*,*41*.*2*_ *= * 67.7, *P* < 0.001) and a post-hoc pairwise comparisons indicated that all three clusters were significantly different from one another (*P* < 0.05 for both δ^13^C and δ^15^N). The three clusters represented adult female SSL that foraged inshore (δ^13^C = -11.9 ± 0.5 ‰, δ^15^N = 20.3 ± 1.0 ‰; mean ± one standard deviation), offshore (δ^13^C = -13.6 ± 0.4 ‰, δ^15^N = 17.7 ± 0.6 ‰) and a cluster best described as being intermediate (mean δ^13^C = -12.6 ± 0.2 ‰, δ^15^N = 18.3 ± 0.8 ‰). Overall, pups that were assigned to the inshore and offshore clusters were equally common (n = 28 or 43% of samples), while the intermediate cluster was less frequent (n = 9 or 14% of samples).

**Table 1:** A total of 65 southern sea lion pup vibrissae samples were analysed from 19 breeding colonies. Vibrissae from Big Shag and Turn Island were collected in 2013 while all other samples were collected in 2014. Also reported are colony size, distance from Patagonian Shelf slope (200 m depth contour) and the proportion of pups assigned to inshore, offshore and intermediate clusters, as determined by a model-based clustering.

Island	Region	Colony size (annual pup production)	Distance from Patagonian Shelf slope (km)	Number of vibrissae analysed	Inshore (%)	Offshore (%)	Intermediate (%)
Sandy Tyssen	East	56	100	1	100	0	0
West Tyssen	East	126	96	1	0	100	0
Big Shag Island	East	328	95	16	38	56	6
Blind Island	East	159	95	1	0	0	100
Cattle Point Island	East	5	76	2	100	0	0
Green Island	East	181	59	5	40	40	20
Motley Island	East	18	43	3	33	33	33
Sal Island	East	83	49	1	0	0	100
Turn Island	East	51	63	16	81	13	6
North Fur Island	West	39	93	1	0	100	0
South Fur Island	West	3	110	2	0	100	0
Twins North	West	49	100	2	0	100	0
Twins South	West	68	100	2	0	100	0
Circum Island	West	71	49	1	100	0	0
Outer Island	West	45	63	1	0	0	100
Peat Island	West	39	35	1	0	100	0
Stinker Island	West	135	46	3	0	100	0
Tussock Island	West	71	38	4	25	50	25
West Double Creek	West	34	50	2	50	0	50
	**Total**						
	East			46	54	33	13
	West			19	16	68	16

Detailed characterisation of colony-level differences in stable isotope ratios was not possible because most of the colonies were represented by one or only a handful of samples. However, a significant difference between West and East Falkland in the overall proportion of pups assigned to the inshore and offshore clusters was detected (χ^2^ = 8.8, *n* = 56, *P* = 0.003), with a larger proportion of pups being assigned to the offshore cluster in West Falkland (Table 1). To explore this further, we tested whether the relative proportion of pups assigned to inshore and offshore clusters at a given colony varied with distance from the Patagonian Shelf slope. No significant effect of distance was found (χ^2^_1,14_ = 2.6, *P* = 0.11), suggesting that proximity to the Patagonian Shelf slope does not explain geographic variation in adult female SSL habitat use.

Finally, in order to assess differences in foraging behaviour at the colony level, we focussed on the two colonies for which we had the largest sample sizes, Big Shag Island and Turn Island, both of which are in East Falkland. We found that a significantly larger proportion of pups were assigned to the offshore cluster at Big Shag relative to Turn Island (56% versus 13% respectively; Table 1; χ^2^ = 7.0, *n* = 30, *P* = 0.008). Again, this difference was not consistent with expectations based on proximity to the Patagonian Shelf slope, as Turn Island was closer to the slope yet had a larger proportion of pups assigned to the inshore cluster. We did not detect intersexual differences in δ^13^C or δ^15^N at Big Shag Island or Turn Island (Welch two sample t-test, *t* < 1.1, *P* > 0.05 for all comparisons).

## Discussion

By collecting vibrissae from pups at 19 different breeding colonies, we characterised the habitat use of adult female SSL during gestation across their geographical range at the Falkland Islands. Sample sizes from individual colonies were often small because logistical constraints prevented extended visits. Nevertheless, the overall spatial scale of sampling was unprecedented for this species. Our data showed that pup vibrissae captured the same inshore and offshore pattern as previously described for adult female SSL breeding at Big Shag Island on the basis of biologging data and stable isotope analysis of adult female vibrissae [16]. A broadly similar pattern in habitat use is also reported in Argentina, where adult female SSL are suggested to shift from offshore pelagic prey to coastal benthic prey between pre- and post-parturition [22]. In contrast, the inshore and offshore pattern in adult female habitat use that we described is unlikely to reflect a shift in habitat use from pre- to post-parturition or be an artefact of pup age, because we analysed segments of pup vibrissae that were grown while the pup was *in utero*.

Efficiency trade-offs may help to explain why certain adult female SSL foraged offshore, whilst others presumably reduce their travel costs and foraged inshore [16]. If we assume that individuals act to maximise their net rate of energy intake, then optimal foraging theory suggests that individuals should ignore certain types of prey that are less profitable and invest time searching for more valuable prey [23–25]. For example, offshore pelagic prey in northern Patagonia have a higher energy density and lipid content than coastal benthic prey, suggesting offshore foraging trips maybe rewarded with higher energy prey [18]. However, in other marine mammal species factors such as body size, age and experience influence prey handling, search efficiency or dietary preference [26–28]. Hence, intrinsic factors likely shape individual habitat use and might explain why individuals differed in their preference for inshore or offshore habitats.

Despite sample sizes at individual breeding colonies generally being small, our results suggested that adult female SSL used both inshore and offshore habitats across the geographic range of this species at the Falkland Islands. However, the proportion of pups assigned to the inshore and offshore clusters appeared to differ between West and East Falkland. Taken at face value, this implies broad-scale geographical variation in foraging strategies between West and East Falkland. However, sample size was too small to control for data dependency resulting from repeated measures of pups from the same breeding colony, nested within West and East Falkland. Hence, larger sample sizes are ultimately needed to confirm the difference between West and East Falkland.

Analysis of the two colonies from which the largest sample sizes could be collected identified significant colony-level differences in the use of inshore and offshore habitats. The exact reasons for this remain unclear, but pups were sampled at random, suggesting that there would have to be marked differences among colonies in the size and age distribution of reproductively active females for intrinsic factors to explain the variation in habitat use. Given that no size differences were evident between adult female SSL that foraged inshore or offshore at Big Shag Island [16], an alternative explanation may be that differences in habitat use reflect the proximity of breeding colonies to prey resources.

The Patagonian Shelf slope is a predictable oceanographic feature and a region associated with enhanced biological activity and productivity [29]. Given foraging efficiency is linked to individual fitness, it might be reasonable to assume that, when colonies are in close proximity to the Patagonian Shelf slope, a larger proportion of adult female SSL forage offshore, where foraging efficiency maybe enhanced by aggregations of prey such as cephalopods [30,31]. However, pups assigned to the inshore cluster were in the majority at Turn Island, whereas at Big Shag Island pups assigned to the offshore cluster were more common, despite the former being closer to the Patagonian Shelf slope. Similarly, in the northwest Falkland Islands, pups were only assigned to the offshore cluster despite these colonies being some of the furthest from the Patagonian Shelf slope. Thus, our data do not support a simple explanation based on proximity of breeding colonies to the Patagonian Shelf slope.

Another prominent oceanographic feature around the Falkland Islands is the Falkland Current. The Falkland Current originates from the Antarctic Circumpolar Current and branches into two main northward flowing currents when it reaches the continental shelf to the south of the Falkland Islands [29]. The eastern branch of the Falkland Current runs along the Patagonian Shelf slope and is associated with meso-scale fronts, quasi-stationary eddies and regions of upwelling [31,32]. The weaker western branch is also associated with quasi-stationary eddies and regions of upwelling, but bottom topography, water structure and the seasonal abundance of fish and squid varies around the Falkland Islands [31–33]. It is possible that colony-level differences in adult female SSL habitat use (and differences between West and East Falkland) reflect differences in the proximity of colonies to predictable oceanographic features, which may in turn influence the abundance, distribution and availability of preferred prey. Given that Big Shag Island has a pup production over five times that of Turn Island, an alternative explanation is that population size could have contributed towards differences in the prevalence of inshore and offshore habitat use if there was density dependant depletion of resources around breeding colonies (e.g., [34]). To distinguish between these possibilities it would be necessary to gather detailed data on local oceanography, SSL diet and patterns of prey availability, which are currently lacking.

Finally, we also found evidence for a third cluster that was intermediate to the inshore and offshore clusters. As our analysis integrated roughly one month of dietary information, the intermediate cluster may have represented adult female SSL that either shifted foraging habitat from inshore to offshore (or *vice versa*) or alternated between habitats. Alternatively, the intermediate cluster could reflect a separate strategy, such as a predominantly benthic or pelagic foraging mode, as previously described for adult female SSL breeding at the Falkland Islands that foraged offshore [16]. A more detailed understanding of the foraging behaviour associated with the intermediate cluster may yield further insights into individual specialization in SSL and represents an interesting avenue of research for future studies.

In conclusion, although our study provides only a temporal snapshot, the spatial range of sampling allowed us to obtain a broader and more detailed picture of SSL foraging ecology than was previously possible. Our results not only support a previous study showing that adult female SSL utilize at least two distinct foraging habitats, but we also showed that pups assigned to the three clusters (inshore, offshore and intermediate) were present at breeding colonies across the Falkland Islands. Additionally, we found appreciable variation in inshore and offshore habitat use between breeding colonies. This observation merits further investigation because it could suggest that different breeding colonies may be differentially impacted by anthropogenically driven habitat modifications depending on the extent to which breeding females utilise inshore and offshore habitats.

## Supporting Information

S1 Fig Uncertainty plots derived from the model-based clustering of southern sea lion pup stable isotopes, implemented within the R Package ‘Mclust’. The ellipses shown represent each cluster, with the smallest grey dots representing the lowest uncertainty and large black dots, the largest uncertainty (quantiles are 0.75,0.95 –the default quantiles used in the ‘Mclust’ package).
(DOCX)

S1 Table Isotope data for 65 southern sea lion pups sampled from breeding colonies around the Falkland Islands (see ‘Methods’ for details). (DOCX)
