# Supplementary material for: Pup Vibrissae Stable Isotopes Reveal Geographic Differences in Adult Female Southern Sea Lion Habitat Use during Gestation
Source: PLoS One. 2016 Jun 15;11(6):e0157394. doi: 10.1371/journal.pone.0157394 (PMC4909279; doi:10.1371/journal.pone.0157394)
Supplement: S1 Fig — The ellipses shown represent each cluster, with the smallest grey dots representing the lowest uncertainty and large black dots, the largest uncertainty (quantiles are 0.75,0.95 –the default quantiles used in the ‘Mclust’ package). (DOCX) [file pone.0157394.s001.docx]

**Supporting information to:**

**Pup vibrissae stable isotopes reveal geographic differences in adult female southern sea lion habitat use during gestation**

Alastair M. M. Baylis, Gabriele J. Kowalski, C. C. Voigt, Rachael A. Orben, Fritz Trillmich, Iain J. Staniland, Joeseph I. Hoffman

**Content:**

**S1 Fig: Uncertainty plots derived from the model-based cluster analysis of southern sea lion pup stable isotopes, implemented within the R Package ‘Mclust’.** The ellipses shown represent each cluster, with the smallest grey dots representing the lowest uncertainty and large black dots, the largest uncertainty (quantiles are 0.75,0.95 – the default quantiles used in the ‘Mclust’ package).


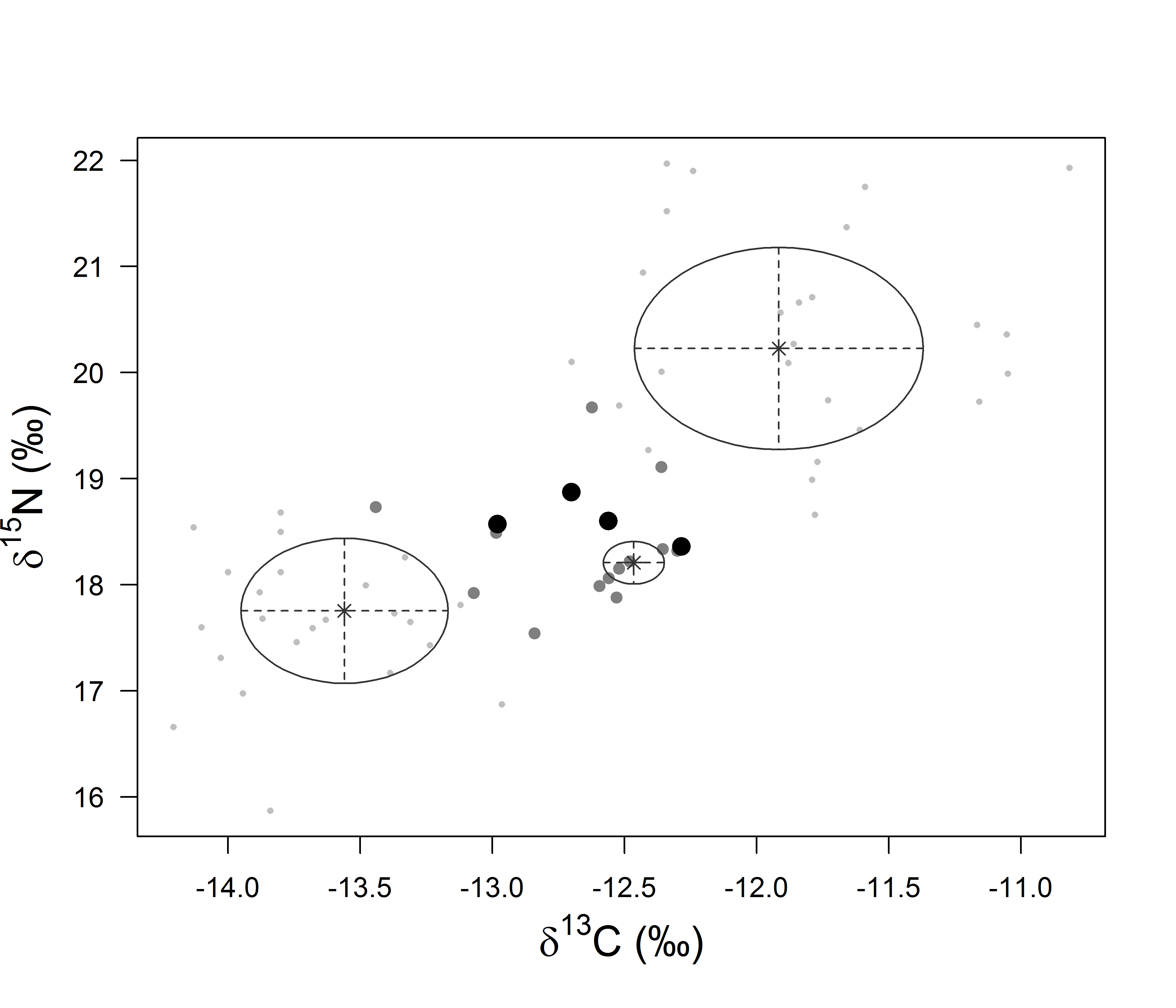


S1 Fig
