# Supplementary material for: Pup Vibrissae Stable Isotopes Reveal Geographic Differences in Adult Female Southern Sea Lion Habitat Use during Gestation
Source: PLoS One. 2016 Jun 15;11(6):e0157394. doi: 10.1371/journal.pone.0157394 (PMC4909279; doi:10.1371/journal.pone.0157394)
Supplement: S1 Table — (DOCX) [file pone.0157394.s002.docx]

**Supporting information to:**

**Pup vibrissae stable isotopes reveal geographic differences in adult female southern sea lion habitat use during gestation**

Alastair M. M. Baylis, Gabriele J. Kowalski, C. C. Voigt, Rachael A. Orben, Fritz Trillmich, Iain J. Staniland, Joeseph I. Hoffman

**Content:**

**S1 Table: Isotope data for 65 Southern sea lion pups sampled from breeding colonies around the Falkland Islands (see ‘Methods’ for details).**

S1 Table

| **δ^13^C** | **δ^15^N** | **Island** |
| --- | --- | --- |
| -12.6 | 19.7 | West Double Creek |
| -11.8 | 18.7 | Tussock Island |
| -11.2 | 19.7 | Circum Island |
| -11.1 | 20.0 | Green Island |
| -11.1 | 20.4 | Cattle Point Island |
| -10.8 | 21.9 | Cattle Point Island |
| -11.8 | 19.0 | Green Island |
| -11.2 | 20.5 | Sandy Tyssen |
| -12.7 | 20.1 | Big Shag Island |
| -11.7 | 19.7 | Big Shag Island |
| -11.7 | 21.4 | Big Shag Island |
| -11.8 | 20.7 | Big Shag Island |
| -12.5 | 19.7 | Big Shag Island |
| -11.9 | 20.3 | Big Shag Island |
| -12.4 | 19.3 | Turn Island |
| -11.8 | 20.7 | Turn Island |
| -12.2 | 21.9 | Turn Island |
| -12.3 | 22.0 | Turn Island |
| -11.9 | 20.6 | Turn Island |
| -11.8 | 19.2 | Turn Island |
| -12.4 | 20.9 | Turn Island |
| -11.6 | 19.5 | Turn Island |
| -12.4 | 20.0 | Turn Island |
| -11.6 | 21.8 | Turn Island |
| -11.9 | 20.1 | Turn Island |
| -12.3 | 21.5 | Turn Island |
| -12.4 | 19.1 | Motley Island |
| -13.4 | 17.2 | Stinker Island |
| -13.8 | 18.7 | Stinker Island |
| -14.0 | 17.3 | Tussock Island |
| -13.3 | 17.7 | Tussock Island |
| -13.9 | 17.7 | Stinker Island |
| -14.2 | 16.7 | Twins North |
| -13.7 | 17.6 | North Fur Island |
| -13.5 | 18.0 | South Fur Island |
| -13.1 | 17.8 | Twins North |
| -13.2 | 17.4 | South Fur Island |
| -13.9 | 17.0 | Twins South |
| -13.8 | 15.9 | Green Island |
| -12.8 | 17.5 | Green Island |
| -13.1 | 17.9 | West Tyseen |
| -13.0 | 16.9 | Peat Island |
| -13.8 | 18.5 | Big Shag Island |
| -13.9 | 17.9 | Big Shag Island |
| -14.1 | 17.6 | Big Shag Island |
| -14.1 | 18.5 | Big Shag Island |
| -14.0 | 18.1 | Big Shag Island |
| -13.7 | 17.5 | Big Shag Island |
| -13.8 | 18.1 | Big Shag Island |
| -13.6 | 17.7 | Big Shag Island |
| -13.4 | 18.7 | Turn Island |
| -13.4 | 17.7 | Turn Island |
| -13.3 | 18.3 | Motley Island |
| -12.4 | 18.3 | West Double Creek |
| -12.5 | 18.2 | Tussock Island |
| -13.0 | 18.5 | Twins South |
| -12.6 | 18.1 | Outer Island |
| -12.6 | 18.0 | Green Island |
| -12.5 | 17.9 | Sal Island |
| -12.3 | 18.4 | Blind Island |
| -12.3 | 18.3 | Big Shag Island |
| -13.0 | 18.6 | Big Shag Island |
| -12.7 | 18.9 | Turn Island |
| -12.6 | 18.6 | Turn Island |
| -12.5 | 18.2 | Motley Island |
